# Gasdermins assemble; recent developments in bacteriology and pharmacology

**DOI:** 10.3389/fimmu.2023.1173519

**Published:** 2023-05-17

**Authors:** Claudine S. Greenwood, Meghan A. Wynosky-Dolfi, Allison M. Beal, Lee M. Booty

**Affiliations:** ^1^ Chemical Biology, GSK, Stevenage, United Kingdom; ^2^ Pure and Applied Chemistry, University of Strathclyde, Glasgow, United Kingdom; ^3^ Immunology Research Unit, GSK, Philadelphia, PA, United States; ^4^ Immunology Network, GSK, Stevenage, United Kingdom

**Keywords:** gasdermin, pyroptosis, bacteriolgy, pharmacology, inflammasome

## Abstract

The discovery of gasdermin D (GSDMD) as the terminal executioner of pyroptosis provided a large piece of the cell death puzzle, whilst simultaneously and firmly putting the gasdermin family into the limelight. In its purest form, GSDMD provides a connection between the innate alarm systems to an explosive, inflammatory form of cell death to jolt the local environment into immunological action. However, the gasdermin field has moved rapidly and significantly since the original seminal work and novel functions and mechanisms have been recently uncovered, particularly in response to infection. Gasdermins regulate and are regulated by mechanisms such as autophagy, metabolism and NETosis in fighting pathogen and protecting host. Importantly, activators and interactors of the other gasdermins, not just GSDMD, have been recently elucidated and have opened new avenues for gasdermin-based discovery. Key to this is the development of potent and specific tool molecules, so far a challenge for the field. Here we will cover some of these recently discovered areas in relation to bacterial infection before providing an overview of the pharmacological landscape and the challenges associated with targeting gasdermins.

## Introduction

The discovery of pyroptosis is inherently linked to bacterial immunology. Early studies of macrophages lysing in a fiery demise after exposure to the lethal toxin of anthrax were, at the time, unappreciated observations of pyroptosis (1, 2). The interaction between the *Shigella flexneri* protein ipaB and caspase-1 in macrophages led to the first designations of pyroptosis; typically characterised by release of pro-inflammatory cytokines through pores consisting of N-terminal oligomers of one of the gasdermin protein family, followed by inflammatory cell death (3, 4). The history of pyroptosis and the historical context of bacterial-gasdermin interactions have been extensively covered elsewhere (5–9) and so here we will instead focus on bringing together the very recent discoveries in the context of bacteriology and gasdermins, before discussing the pharmacological landscape and associated challenges.

**Figure 1:**
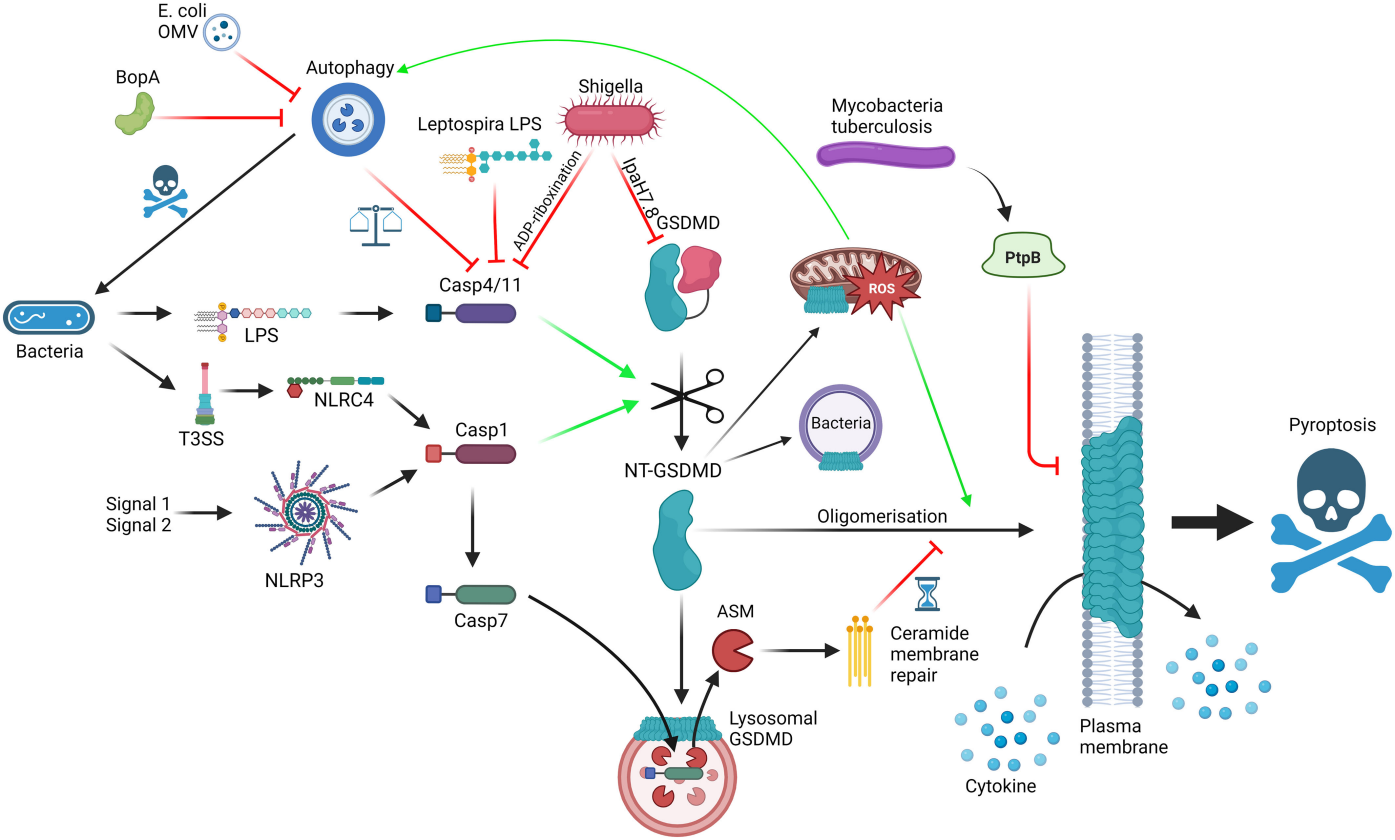
Interactions between GSDMD, the cellular machinery and bacteria; GSDMD can be activated *via* LPS detection by caspase-4/11 (non-canonical inflammasome) or NLRP3/NLRC4 activation (canonical inflammasome), leading to cleavage of FL-GSDMD into N-terminal GSDMD, which can oligomerise and form pores on the plasma membrane, mediating cytokine release and pyroptosis. Mechanisms to slow down pyroptosis include caspase-7-ASM mediated ceramide-based membrane repair and autophagy-mediated harbouring of caspase-4/11. GSDMD pores can bind to lysosomes, allowing caspase-7 entry, and mitochondria, promoting ROS, which promotes autophagy and GSDMD oligomerisation. GSDMD can also have direct bactericidal activity. Bacteria can themselves interact with the pathway using T3SS virulence factors such as Ipa7.8-mediated degradation of GSDMD, ADP-riboxination of caspase-4/11, alternative LPS to limit caspase-4/11 dimerisation, BopA and outer membrane vesicle (OMV) inhibition of autophagy and PtpB dysregulation of membrane phosphorylation and stability. Red = inhibitory; Green = activating. Made with BioRender.

Briefly, the gasdermin family consists of six proteins in human (GSDMA, GSDMB, GSDMC, GSDMD, GSDME and GSDMF/PJVK) and ten proteins in mouse (GSDMA1-3, GSDMC1-4, GSDMD, GSDME, PJVK) (9). All except GSDMF have been shown to exhibit pyroptotic functionality *via* the N-terminal pore-forming domain, which exhibits lipid preferences depending on the family member (10). Since the discovery of GSDMD downstream of caspase-11 (4/5 in human) by two independent groups (11, 12), an explosion of mechanisms that drive gasdermin activation and inhibition have been uncovered. The first characterisation of GSDMD activation and induction of pyroptosis was through cytosolic LPS-induced caspase-11 cleavage of GSDMD at Asp275 and release of the C-terminal domain (**Figure 1**). This leads to the oligomerisation of consequently uninhibited N-terminal fragments that traverse to and insert in the plasma membrane and form a pore in the cell, resulting in cytokine release and eventual cell death through lysis (11, 12). It was recently discovered that gasdermins are required for the release of IL-1 family cytokines (IL-1α, IL-1β and IL-18), but an additional protein NINJ1 is critical for the plasma membrane rupture (PMR) and HMGB1 release characteristic of inflammasome activation (13). Through mouse studies, it has been shown that both cytokine release as well as cell death is important for anti-bacterial host defence to pathogens such as *Citrobacter rodentium* (13, 14). This all occurs rapidly and without the usual organisation of apoptosis, leading to cellular contents flooding, and consequently activating, the local environment to flag immune cells to the location of danger (15–18).

Since these seminal discoveries, the functions, regulation, and consequences of gasdermin activation and/or inhibition has expanded considerably. Just focussing on GSDMD, there is a now a well-established contribution from inputs other than inflammatory caspases. Apoptotic caspases such as caspase-3 and caspase-8 have emerged as inflammasome-independent contributors to gasdermin activation (19). Caspase independent contributions to gasdermin function also exist in the form of cathepsins (20, 21), granzymes (22, 23) and elastase, neutrophil expressed (ELANE) (24, 25). These different drivers have both distinct and overlapping biological consequences dependent on the cellular context and/or sensed DAMP/pathogen, including switches between pyroptosis to apoptosis and necroptosis. There are emerging examples of virulence factors interfering with gasdermin function including targeting GSDMB and GSDMD for proteasomal degradation (26–28). Additionally, subcellular organelle-gasdermin interactions exist including in the mitochondria (29–32), endoplasmic reticulum (33), autophagosome (33) and the nucleus (34) suggesting that subcellular localization patterns may regulate biological functions. Moreover, non-pore forming functions have emerged including full length GSDMB regulating proliferation, migration and adhesion in the context of the gut (35), full length GSDMD mediating the homeostasis of intestinal epithelium and control of IL-1β containing extracellular vesicles in an inflammasome, activation-independent function (36), as well as mucus secretion and barrier integrity (37), reviewed in (38). This review will bring together canonical pyroptotic functions of the gasdermin family with these alternative activation options, non-pyroptotic elements and subcellular interactions within the framework and context of bacterial infection and immunity.

## GSDMD

As the pyroptosis pioneer, GSDMD is by far the best characterised gasdermin of the family. One major advantage to studying GSDMD is in the simplicity of driving inflammasome activation with clear assay-amenable cellular responses. Inflammasomes were defined in 2002 (39) and have well established *in vitro* and *in vivo* assays with clear end points, that we now appreciate as, at least partially, GSDMD-mediated. It is entirely possible, like in the case for GSDMB (23), we are already using stimulation conditions of lesser-understood gasdermin activation but are currently unaware of the gasdermin link. Now the field has elucidated some activation conditions for the pore forming behaviours of the other gasdermins, these authors wait with anticipation for the next wave of studies with the lesser understood gasdermins. Given the exciting roles emerging in bacteriology and disease, we suspect gasdermins have a lot more to teach us including GSDMD.

GSDMD’s pyroptotic potential was uncovered by two complementary studies in 2015, one using an *in vivo* forward genetic screen (11) and the other an *in vitro* CRISPR-Cas9 screen in mouse bone marrow derived macrophages (BMDM) (12). In both cases, GSDMD emerged as a key player in the lipopolysaccharide (LPS)-induced non-canonical inflammasome model deployed by both studies. Since, the family (except GSDMF) were shown to possess N-terminal pore-forming and C-terminal autoinhibitory functions (10), and GSDMD specifically has been shown to be activated by other caspases, in both activation and inhibition, as well as by other proteases (19–21, 24). It should be noted that GSDMB can engage lipid binding *in vitro*, even in the presence of the C-terminal domain, indicating a possible divergent role associated with lipid interactions for full length GSDMB (40). Like the first *in vitro* screen, most of the GSDMD work to date has focussed on its function in macrophages, considered to be most relevant for pyroptotic function. In addition to cellular pyroptosis, GSDMD also possess direct bactericidal activity (41) as well as functions independent from pyroptosis. Here, we will cover those most relevant to bacterial infection contexts, with a particular focus on those that would benefit from modulation by pharmacological intervention highlighting the potential in GSDM-based therapies.

## Autophagy and pyroptosis – a delicate balance

One area of particular interest is the interplay between autophagy and pyroptosis, and how GSDMD is involved to carefully balance host response to pathogenicity of invader, and how invaders inhibit these pathways to shift the balance in their favour (**Figure 1**). Although covered excellently by Harvest and Miao (42), who also provide an interesting hypothesis to legitimise the relationship between autophagy and pyroptosis, it is worth briefly covering some of the key findings in the context of this review. As covered above, GSDMD connects direct pathogen sensing to pyroptotic response. This occurs due to the exposure of bacterial LPS by IFNγ-induced guanylate-binding proteins (GBP) to create a docking site and activation platform on the bacteria for caspase-4, leading to GSDMD cleavage and activation, thereby protecting the host from pathogen replication (43). Although incredibly effective, pyroptosis is also potentially dangerous to the host and, as such, evolution has had to consider the relevance of pyroptosis to the invading pathogen, the benefit/risk ratio in the tissue niche and the ability of virulence factors to limit weapon potency. *S. flexneri*, a commonly used model system as covered above, is incredibly pathogenic requiring very low numbers of invading bacteria to colonise and cause shigellosis, primarily a diarrheal condition with high prevalence in young children (44). *S. flexneri* uses its Type 3 Secretion system (T3SS) and subsequent injection of virulence factors into cells to invade and infect the host. In the context of pyroptosis, *S. flexneri* uses Osc3C to inhibit caspase-4/11, avoiding the LPS-detection from the non-canonical inflammasome (45–47). To do this, *S. flexneri* deploys a covalent modification of caspase-4 and 11 *via* ADP-riboxination of Arg314 and Arg310, respectively, inhibiting caspase autoprocessing and pyroptotic response. In doing so, *S. flexneri* has exposed itself to the T3SS-detection system of NAIP/NLRC4 but *S. flexneri* is one step ahead, through Ipa7.8H-mediated degradation of GSDMD (and GSDMB) directly, rendering NAIP-NLRC4 without pyroptotic bite (27, 28, 48). These virulence factors likely explain the high pathogenicity of *S. flexneri* whereas in the case of *Burkholderia thailandensis*, another Gram-negative bacteria possessing a Shigella-like T3SS, humans and mice are extraordinarily resistant to infection. This may be due to the activities of caspase-4/11 and GSDMD, with neutrophilic pyroptosis as the main tool to remove *B. thailandensis* infection (49, 50). Interestingly, a closely related pathogen *Burkholderia cepacia* lacks the highly pathogenic Shigella-like T3SS but can still invade the cytosol and activate caspase-11 (49). The cytosolic presence of *B. cepacia* also activates autophagy in a caspase-11/GSDMD dependent manner, leads to GSDMD-mediated mitochondrial dysfunction and enhances reactive oxygen species (ROS)-induced autophagy, and ultimately restricting the growth of the bacterium without driving pyroptosis (51–54). The balance between pyroptosis and autophagy seems quite contrasting, yet Harvest and Miao present an excellent hypothesis suggesting that autophagy directly deals with intracellular bacteria without sacrificing the host, whereas pyroptosis deals with the infection by recruitment of local phagocytes and immune components (42). Through an intricate balance of GSDMD-mediated autophagy activation coupled with self-capture of caspase-11 and subsequent limiting of GSDMD-driven pyroptosis, the cell can carefully remove the pathogen without committing explosive suicide, with any remaining pores rescued by the repair machinery (55). The cell can use this rheostat to respond accordingly to the level of pathogenicity in the invader. For example, *B. thailandensis* possess BopA, an inhibitor of autophagy. Therefore, the cell cannot sequester the activated caspase-11 *via* autophagy and so, in the absence of inhibitory GSDMD virulence factors, the cell commits to pyroptosis and clears the pathogen at the sacrifice of the host cell. In an opposite way, *Escherichia coli* outer membrane vesicles possess autophagy inhibitors so the macrophage engages the non-canonical inflammasome instead (56). At the extreme end of pathogenicity, *S. flexneri* is able to inhibit caspase-11, GSDMD and autophagy, meaning the cell has limited options and thus explains the lack of innate immunity against *S. flexneri*. We highly recommend a detailed insight into the processes and proposed mechanisms in the focussed review for a deeper dive into the autophagy-pyroptosis axis in control of bacterial infection (42).

Bacteria deploy a wide range of pyroptosis-interfering virulence factors in an attempt to subvert innate immunity through disrupting gasdermin-mediated response. *Mycobacterium tuberculosis* deploys PtpB, a phospholipid phosphatase capable of dephosphorylating phosphatidylinositol-4-monophosphate and phosphatidylinositol-(4,5)-bisphosphate and as such disrupting GSDMD pore formation in the membrane (57). This relies on PtpB to hijack ubiquitin to activate the dephosphorylation function. Mutating either the phosphatase function or the ubiquitin binding site of PtpB renders *M. tuberculosis* vulnerable to attack. In another attempt to subvert immunity *Leptospira interrogans*, the bacteria responsible for leptospirosis, possesses an atypical LPS to avoid detection by caspase-4/11 (58). It is so different in fact that it can actively antagonise caspase-4/11 from detecting LPS from other organisms, such as *E. coli* (**Figure 1**). Consistent with this mechanism, the authors found that IL-1R plays little role in regulating *L. interrogans* growth *in vivo*, highlighting another strategy to subvert the innate immune pathways relevant to avoiding GSDMD-mediated pyroptosis. As the field uncovers more about the detailed molecular mechanisms controlling the intricate rheostat which balances protective GSDMD-mediated responses as described above without full blown unrestrained pyroptosis, we gain the puzzle pieces that will enable the design of small molecules to selectively modulate this process and boost host defences against pathogens which aim to subvert them.

## Metabolic and redox control of GSDMD

Evidence for internal rheostats to control the commitment to full pyroptosis depending on pathogenicity and cellular state is building. Recent observations in this context around metabolic control of pyroptosis, and specifically the contribution of mitochondrial ROS, are of interest in the context of bacterial infections. The Ragulator-Rag complex is known to regulate mTORC1 activation through trafficking to the lysosome and links amino acid sensing to the downstream signalling of mTOR, a major cell signalling node (59). Two groups used CRISPR-Cas9 *in vitro* screening approaches to implicate Ragulator-Rag to GSDMD function and control of pyroptosis (29, 60). Evavold et al. used engineered BMDMs in a forward genome wide screen to find regulators of GSDMD oligomerisation in an inducible N-terminal mediated pore formation model (29), whereas Zheng et al. compared LPS electroporation versus LPS + TAK1 inhibitor to replicate *Yersinia pestis* infection, the pathogen that causes plague, in a genome wide screen (60). In both cases subunits of the Rag-Ragulator complex were identified as screen hits. Together the studies highlight the requirement for Rag-Ragulator in GSDMD oligomerisation and pyroptotic response to *Yersinia*, through caspase-8. In addition, Evavold et al. found that addition of electron transport chain inhibitors enhanced GSDMD oligomerisation in a Ragulator-Rag independent fashion, and addition of N-acetyl cysteine (NAC) blocked such response, implicating mitochondrial dysfunction and specifically mitochondrial ROS. The lack of requirement for Ragulator-Rag in this model suggests the role of Ragulator-Rag might be to drive mitochondrial dysfunction and ROS production to enhance GSDMD oligomerisation and consequently pyroptosis, as has been previously observed (61). Building on this, a recent observation elucidated cysteine 192 of murine GSDMD (cysteine 191 in human GSDMD) to be critical in sensing mitochondrial ROS to promote oligomerisation and goes some way to explain the inhibitory effect of C192-targeting disulfiram (62, 63). In Zheng et al., there is also evidence of a direct engagement between Rag-Ragulator, FADD, RIPK1 and caspase-8, and suggestion that mTORC1 is not necessary for pyroptosis (60).

More evidence is available to strengthen the connection between metabolic homeostasis and gasdermin function. In the case of LRRK2^G2019S^ gain of function mutations, macrophages exhibit considerable mitochondrial dysfunction, mediated by GSDMD-mitochondria binding driving a switch from pyroptosis to necroptosis, mediated by RIPK1/RIPK3/MLKL (32). What isn’t clear is why GSDMD preferentially selects mitochondrial membranes in this scenario, but it is known that LRRK mutations drive changes in the mitochondrial structure, dynamics and ER-tethering as well as lipid dysfunction, which may increase the possibility of GSDMD-mitochondrial binding over the plasma membrane (64–66). Relevant to this review though is the consequence on infection. Particularly interesting is that this mechanism is evolutionary conserved as the authors show both LRRK2^G2019S^ expressing flies infected with *Pseudomonas entomophila* and LRRK2^G2019S^ mice infected with *Mycobacterium tuberculosis* exhibit hyperinflammation, higher bacterial burden and enhanced immunopathology (32). This mitochondrial ROS-GSDMD axis is also seen to be important in surface expression of the synovial T cell receptor-γδ ligand on CD14^+^CD16^+^ monocytes, in conjunction with toll-like receptor (TLR) stimulation, mitochondrial metabolism and inflammasome activation in a redox sensitive manner (67). This is important in regulating the response of γδ T cells in infection and autoimmunity, where overactive GSDMD may contribute to overexpression of surface γδ ligand, driving aberrant adaptive immune response, and targeting GSDMD at the mitochondrial ROS-GSDMD axis in inflammatory monocytes may provide a useful approach. Indeed, targeting GSDMD in any of the above scenarios may provide an alternative approach to targeting metabolism or mitochondrial dysfunction which have their challenges as pharmacological platforms. The opposite is also true whereby modulating autophagy, metabolism or redox balance is an opportunity to control GSDMD function, and ultimately pyroptotic response. The authors await studies that further detail the molecular processes and biophysical properties that control GSDMD function to selectively modulate these processes with knowledge-based drug design.

## Strategies for host protection

As above, examples of host cells dialling back pyroptosis to limit cellular damage whilst ensuring a robust immune response are clear. These observations highlight the need for cells, particularly at barrier sites, to flag the invasion to neighbouring immune cells without compromising barrier integrity and exacerbating any pathology. Although not bacterial, *Entamoeba histolytic* implicates a similar inflammasome-gasdermin response in the gut upon infection (68). Most typically *E. histolytica* infects the gut of a host while the host remains asymptomatic. However, in some individuals the pathogen can break through the gut and drive inflammation. Due to its size, *E. histolytica* is detected by macrophages through a Gal-lectin surface adhesin, which activates the NLRP3 response and recruits the immune system *via* caspase-4 and GSDMD, potentiated by ROS and K^+^ efflux (68). In addition, *E. histolytica* infected macrophages downregulate NINJ1, a key cell lysis regulatory protein, coupled with processing of GSDMD and IL-1β *via* caspase-1 and caspase-4 (13, 69). This culminates in a hyperactivated macrophage, with active GSDMD pores and cytokine release, but no cell lysis. It is not clear what mechanisms control NINJ1 regulation in this context but understanding this may provide opportunities to control damaging NINJ1-mediate cell lysis driven pathology associated with over-inflammation such as sepsis.

Controlling GSDMD contribution to pyroptosis is important in the case of Streptococcal infections*. Streptococci* are a Gram-positive, typically diplococci bacterial genus that exist mostly symbiotically with hosts, except a number of strains that are major causes of infections (70). Pathogenic *Streptococci* cause mild to severe disease ranging from strep throat to meningitis and pneumonia and are a major socioeconomic burden globally. The emergence of antimicrobial resistant strains has accelerated the need for new therapies in this space to tackle associated pathology, such as sepsis. Although GSDMD has been linked to protecting against some *Streptococci* infections (71), failure to control infection and the subsequent inflammation, upon which gasdermins play a critical role, can lead to sepsis and death. GSDMD has been linked to accelerating sepsis by providing a conduit for passive release of both SQSTM1, a regulator of innate immunity, and F3, a blood coagulation initiator downstream of LPS or STING activation, respectively (72, 73). In both cases, pharmacological interference of the mediating pathways was beneficial, highlighting the potential for GSDMD as a legitimate target in this therapeutic space. There are also examples of evolution-directed mechanisms to limit gasdermin function in *Streptococci* infection. As will be covered in more detail later, recent seminal studies have uncovered a protective function of keratinocyte GSDMA in response to Group A Streptococcal protease SpeB (74, 75) (see GSDMA section below). As for GSDMD and GSDME, the cytokine IL-6 has been proposed to have a protective role in lung pneumococcal infections (76). In this case, IL-6 exhibited an inhibitory effect on GSDMD and GSDME mediated pyroptosis in lung macrophages, preventing cell death and lung injury, through a post-translational modification somewhere along the cascade. It is not clear from this study what the specific mechanism of IL-6 is, nor whether it is acting on GSDMD/E specifically, but the finding suggests a mechanism exists by which host cytokine balance can control pyroptotic flux to manage inflammation and tissue damage. Appreciating this in the context of disease and cytokine dysregulation would be insightful, and defining the specific cytokine-induced modifications could provide a novel approach to control GSDMD/E activity.

In another case of evolved protection against overactive permeable membrane-cell death, Nozaki and Maltez et al. elucidated a role for caspase-7 in eliciting membrane repair in the face of both perforin and gasdermin pore formation (77). Traditionally, caspase-7 was seen to be an ineffective secondary to caspase-3, with no independent function except an intriguing ability to be activated by caspase-1 (78). In this study, the authors noticed correlation between cleaved caspase-7 and the ability of intestinal epithelial cells to extrude upon infection with *Salmonella in vivo*. Without caspase-7, these intestinal cells could not extrude and instead were dysfunctional in morphology and function, and in *Casp7* ^-/-^ organoids GSDMD pore formation occurred much faster than wildtype. Mechanistically, the acid sphingomyelinase (ASM)-driven endocytosis membrane repair process is regulated by caspase-7, driving ceramide production and therefore membrane repair, limiting GSDMD driven cell lysis. Fascinatingly, it was lysosomal GSDMD pores that allows conduit of caspase-7 to ASM, which then generates ceramide and provides a membrane repair process against the forming GSDMD pores. The authors propose caspase-7 evolved to protect against perforin pores, and they prove this in CTL and NK-driven clearance of *Listeria* infections, as well as *Salmonella* and *Chromobacterium violaceum* in IECs and hepatocytes, respectively. However, caspase-7 can also protect against GSDMD pores using this same mechanism. It would be interesting to see whether that mechanism exists across other gasdermins as well. This mechanism provides a method to actively kill invading bacteria, whilst maintaining structural integrity, and therefore limiting inflammation to the invaded cell and not the wider tissue. Thus, a potential application in the context of disease would be realized by acutely enhancing caspase-7 activity in IEC during infection.

## NETosis and GSDMD

Neutrophils are a key player in the fight against invading pathogens. One of the key weapons they possess is the ability to deploy neutrophil extracellular traps (NETs), *via* a process called NETosis (79). Particularly effective against large pathogens, NETs are chromatin based structures that act to trap their prey before killing and, although particularly effective, if dysregulated can contribute to immunopathology (79). Both human and murine neutrophils possess elevated levels of GSDMD and GSDME, and two complementary papers originally highlighted GSDMD in the generation of NETs (25, 80, 81). Chen et al. show that cytosolic LPS or invasion by *Salmonella* or *Citrobacter rodentium* activates GSDMD-mediated NETosis *via* caspase-4/11 non-canonical inflammasome signalling (81), whereas Sollberger and colleagues used a small molecule screen to discover LDC7559, a pyrazolo-oxazepine type compound which inhibits PMA-induced NETosis, mediated by neutrophil elastase (25). Beyond this, there is also evolutionary evidence that GSDME drives pyroptosis-mediated NET formation in zebrafish, and that GSDMD-mediated NET formation is negatively associated with outcome in severe COVID-19 (82, 83). These findings may implicate a role for GSDMD in the formation and propagation of NETs as an immune strategy within the conditions evaluated.

However, very recent observations have called into question the role of GSDMD in NET formation, or more accurately the involvement of GSDMD at distinct stages and contexts of NET formation and NETosis (84–86). Firstly, Chauhan et al. show that GSDMD is indispensable for NETosis induced by PMA, but is important in canonical inflammasome activation of neutrophil pyroptosis (86). This is contrary to observations seen by Sollberger et al. (25). However, since it was discovered, LDC7559 is now known to bind and activate phosphofructokinase, liver (PFKL) and not GSDMD (84). In doing so, LDC7559 actually blocks NADPH oxidase-driven ROS formation and suppresses phagocytic function, including NETosis in a GSDMD-independent fashion (84). Going even further, a recent study from Stojkov et al. claims that NET formation is completely independent of GSDMD and pyroptosis (85). In this study, Stojkov and colleagues test C5a and LPS, both known NET inducers in primed neutrophils, as well as transfected LPS as a non-canonical inflammasome activator and LPS followed by nigericin as an established canonical inflammasome activator. In no scenario did *Gsdmd* ^-/-^ neutrophils form less NETs than wildtype equivalents, asking questions about GSDMD involvement in neutrophil NET formation entirely (87). An interesting observation in neutrophils was that optimal production of IL-1β requires GSDMD, surprisingly in the absence of plasma-membrane pore formation and subsequent pyroptosis. This data suggests that whilst NETosis may not require GSDMD, the associated inflammatory conditions are mediated by GSDMD in neutrophils.

The role of GSDMD during infection is likely cell type and pathogen specific, highlighted well in *Pseudomonas aeruginosa* lung infection models (88). In this case, LPS from *E. coli*, *Klebsiella pneumoniae* and *P. aeruginosa* was evaluated for NET formation in *Clec5a^-/-^, Tlr2^-/-^* and *Tlr4^-/-^* neutrophils. NET formation mediated by *P. aeruginosa* LPS was abrogated only when CLEC5a was removed, yet LPS from *E. coli* and *K. pneumoniae* still had effect. Taking this further the authors were able to show a GSDMD-dependent release of cytokine in alveolar macrophages, yet GSDMD was dispensable in neutrophils for caspase-1 driven NET formation. However, it is not clear whether GSDME could compensate in this context. Additionally, *P. aeruginosa* triggered caspase-1 in both human and mouse neutrophils, which was limited by bacterial exotoxins, in a flagellin-mediated manner (89). Here, NLRC4 was activated in the neutrophils, activating caspase-1 and GSDMD, triggering calcium and peptidyl arginine deiminase 4 (PADI4)-mediated histone citrullination, trafficking of neutrophil DNA into the cytoplasm, but without committing to NET formation. Overall, there is contrasting data in the context of neutrophil NET formation and GSDMD involvement and more clarity is needed to advise on targeting GSDMD in neutrophil-mediated pathology. Caution should be taken around the nomenclature used to define NET formation and whether NETosis has been induced with or without cell death taking place (85). In addition, understanding the effect of NET inducers and their global effect on the neutrophil is important.

## GSDMA

GSDMA is primarily expressed in the upper gastrointestinal tract and the skin (90). Associations of GSDMA with asthma and respiratory pathology largely dominated the early literature on this gasdermin (91–93). Early work to assess the function of GSDMA focused on mitochondrial homeostasis (94). The N-terminal functional domain can regulate mitochondrial homeostasis, specifically *via* mitochondrial oxidative stress and Hsp90/Trap1/Tom70 axis, culminating in mitochondrial permeability transition pore (mPTP) opening and mitochondrial collapse. This response is alleviated by the presence of the autoinhibitory GSDMA C-terminal domain, or pharmacological intervention at the points of ROS, mPTP activation or mitochondrial translocation, and an independent study showed GSDMA has a preference for mitochondrial membranes over plasma membranes (95). Using overexpressed N-terminal GSDMD and GSDMA, there was a large differential between the two with regards to pyroptotic response, measured by propidium iodide and lactate dehydrogenase release assays. To address the caveats that come with overexpression of N-terminal fractions, a chimeric GSDMA-GSDMD protein was generated to enable cleavage by GSDMD activators caspase-1 or 4/5 to release an N-terminal GSDMA fragment. Relative to GSDMD, activated GSDMA N-terminal fragments exhibited delayed cell death, despite being cleaved at similar kinetic rates. There was also a clear abundance of N-terminal GSDMA in the mitochondrial fraction as tracked using a NEON-tag and microscopy, as well as functional data showing mitochondrial dysfunction upon N-GSDMA deposition. These observations are of course intriguing and shed light on the divergence of different gasdermin family members in driving cellular/subcellular consequence, even in a somewhat artificial, but informative, system. Specifically, an endogenous activator of GSDMA has yet to be discovered and so the intracellular activation of GSDMA and the context in which it occurs remains to be elucidated. Another consideration is the bacterial-origin of the mitochondria and what this means for direct GSDMA-bacterial interactions. Understanding a key activator of GSDMA endogenous to cells would be important in both the field of mitochondrial biology and immunology.

Despite the endogenous activator of GSDMA remaining elusive, there has been progress in elucidating exogenous activators of GSDMA. Very recently, two seminal papers have linked GSDMA activation to an exogenous factor, SpeB from *Streptococcus* (74, 75). In keratinocytes, which express elevated levels of GSDMA, the presence of SpeB, a Group A Streptococcus cysteine protease virulence factor, can drive GSDMA mediated pyroptosis. The two studies came to this conclusion from different perspectives yet concluded on the same observation. The LaRock et al. study assessed the cleavage ability of SpeB with all GSDM family members, based on the hypothesis that SpeB can cleave IL-1β, and observed cleaved flag-tagged GSDMA, GSDMC and GSDMD in HEK293 cells (75). Given the skin-site expression of GSDMA in relation to *Streptococcus* infection, this group decided to focus on GSDMA and observed GSDMA can defend against *Streptococcus* mediated skin infections in mice through SpeB-driven GSDMA-mediated keratinocyte pyroptosis. Revisiting GSDMC in this context may also be of future interest. Alternatively, Deng *et al.* focussed on the fact SpeB drives epithelial pyroptosis and sought to understand the key mediators of the model (74). A genome wide CRISPR screen in A431 keratinocytes was performed to further understand the role of GSDMA. A431 cells were electroporated with SpeB, cells enriched in GSDMA sgRNAs were resistance to SpeB-dependent pyroptosis. This was confirmed with follow up studies showing the cleavage site at Gln246 and a preference for acidic lipid membranes (74). Both studies then highlight the sensitivity of GSDMA null mouse models to Group A Streptococcus infections. LaRock et al. used a *Gsdma1* ^-/-^/*Gsdma2* ^-/-^/*Gsdma3* ^-/-^ model whereas Deng et al. focussed on *Gsdma1* ^-/-^ only due to the fact only Gsdma1 and 3 are expressed in the skin, and Gsdma3 does not possess the Gln246 cleavage site. Neither study addresses the mitochondrial observations (discussed above), but this would be of interest in the future, especially given the delayed pyroptotic response compared to GSDMD activators, and relationship between mitochondrial function and immunity.

Overall, early observations of genetic links to disease and tendencies to engage mitochondrial membranes give GSDMA a unique and divergent element relative to GSDMD. Exploiting the unique tendencies such as enhanced mitochondrial membrane engagement may allow preferential targeting of gasdermins to trigger protective host defense. The direct cleavage by an exogenous bacterial protease has shed some light onto the potential anti-bacterial function of GSDMA. Whether an endogenous activator can also drive GSDMA functionality with regards to its N-terminal pore forming domain remains to be seen. However, given the genetic link to disease and divergent functions compared to GSDMD, this would be of considerable interest and a possible therapeutic target in those associated pathologies.

## GSDMB

GSDMB is hypothesized to have arisen from a gene duplication event in the 17q12-21 loci, at the evolutionary level of the synapsid clade with resulting presence of GSDMB in humans and bovine species but not mouse, rat or other non-mammals (96). Before the pore-forming function of gasdermins was discovered, and similar to GSDMA, GSDMB was genetically linked to asthma, ulcerative colitis, Crohn’s disease, and rheumatoid arthritis (93, 97–102). Interestingly, the role of GSDMB in respiratory pathology may also come from an unknown regulatory role *via* arachidonate 5-lipoxygenase (5-LO) and transforming growth factor-β which isn’t related to inducing inflammation or pyroptosis (103), or from the alteration in GSDMB structure caused by disease-associated SNPs (40). A recent study focussing on the inflammatory bowel disease (IBD) role of GSDMB showed that GSDMB is able to regulate epithelial cell proliferation and migration, enhancing the repair process independent of pyroptosis (35). Mechanistically this is linked to a pyroptosis-independent function mediated by GSDMB-dependent enhanced adhesion through focal adhesion kinase (FAK) phosphorylation of platelet-derived growth factor subunit A (PDGFA). This in turn drives vinculin focal adhesion, which is disrupted in disease associated GSDMB SNPs. Outside of inflammatory diseases there is a well-established literature base for the role of GSDMB expression changes and in promoting invasion and metastatic potential in cancer (104–107).

GSDMB shares the typical gasdermin family domains of a pore-forming domain, linker region and autoinhibitory C-terminal section, and can partake in N-terminal driven pyroptosis (10). Uniquely GSDMB prefers to bind phosphoinositides and sulfatides unlike other gasdermin family members (40). There is evidence for endogenous activators of GSDMB, namely caspase-1, 3, 6 and 7 (108), but more recently exogenous activators and interactors relevant to bacterial infection and immunity have emerged.

Granzyme A originating from cytotoxic lymphocytes is able to cleave GSDMB at Lys244 (23). Specifically, GSDMB expression in a HEK293 system was the only gasdermin able to induce NK-mediated cell killing. Mechanistically, the perforin-granzyme mechanism is critical for the induction of cell death in this context, specifically Granzyme A. Low or undetectable levels of GSDMB can be upregulated by IFNγ in a number of cell lines, making them sensitive to pyroptosis (23). Critically, Granzyme A was able to cleave GSDMB *in vivo* with enhanced tumour clearance, highlighting the likely reason many cancers downregulate GSDMB in an oncogenic fashion (106, 107). Considering the role of GSDMB in bacterial infection, recent studies provide evidence that bacteria have evolved mechanisms to shut down this Granzyme A driven mechanism of pyroptosis for immune evasion (28, 48). Much like the tumour example described above, Granzyme A-mediated killing of infected cells is an effective mechanism to remove host cells harbouring pathogens, and it is likely GSDMB plays a key role in driving pyroptosis and/or has direct bactericidal activity to achieve this. Interestingly, *S. flexneri* has developed a T3SS-type virulence factor termed IpaH7.8, which has E3 ligase capabilities (27, 48). IpaH7.8 can actively hijack the host ubiquitin system to ubiquitinate GSDMB marking GSDMB for degradation *via* the proteosome, which also happens to GSDMD (28). This renders the Granzyme A mediated clearance process ineffective, promoting bacterial survival. GSDMB was shown to directly attack the bacteria itself, due to its preference for alternative lipids, and avoid pyroptosis of the whole cell, thereby protecting the epithelial barrier. GSDMD is also affected by IpaH7.8 mediated tagging (28, 48). This is intriguing given that *S. flexneri* had been shown to induce caspase-1 mediated pyroptosis in macrophages previously (109). This is explained by the fact the binding affinity of Ipa7.8H is lower for GSDMD than for GSDMB. If GSDMD is engineered based on structural interaction studies to resemble GSDMB, this restores the high binding affinity (48). It is likely that cell-specific contexts play an important role in the consequence of Shigella and Ipa7.8H effectiveness, for example whilst *S. flexneri* causes caspase-1-mediated pyroptosis in a macrophage, in epithelial systems Ipa7.8H was shown to target GSDMD and tag it for degradation (28). Gasdermins are not the only targets of Ipa7.8H and altogether these mechanisms are deployed by Shigella to avoid cell lysis and loss of the replicating niche and promote bacterial survival. Expansion of these findings to other bacterial strains with E3 ligase-like virulence factors and other gasdermins at infection sites may provide wider insight into the battle between bug and host at the gasdermin level with clinical interest and provide insight to enable drug design to either prevent or promote gasdermin degradation depending on the pathological context.

## GSDMC

GSDMC was first uncovered in the early 2000s in metastatic melanoma and referred to as MLZE (110) and then recognised as a gasdermin and renamed in 2007 as GSDMC (111). GSDMC has a leucine zipper region in the C-terminal domain, which is not found in other gasdermins, suggesting a DNA binding function. Other functions of GSDMC have been proposed, including evidence that gene manipulation of GSDMC limited or exacerbated cell proliferation in colorectal cancer cell lines upon siRNA or overexpression, respectively (112).

With regards to activating cascades and consequences, there is now a body of evidence suggesting that TNF-activated caspase-8 can cleave GSDMC and switch the cell from apoptosis to pyroptosis. This occurred in the context of hypoxia under the driver of PD-L1, which upregulated GSDMC expression, with the TNF generated from tumour associated macrophages (113). Intriguingly however, there are mixed observations on the role of GSDMC in tumour biology. In some cases, enhanced expression of GSDMC promotes tumour aggression and growth, suggesting the pyroptotic function of GSDMC is not a critical determinant of its ability to influence tumour homeostasis. To further complicate the role of GSDMC in tumour biology, α-ketoglutarate (KG) has been shown to drive caspase-8 mediated activation of GSDMC and subsequent pyroptosis in cancer cell lines and mouse models. Here, GSDMC expression was correlated to a reduction in tumour growth and volume, suggesting α-KG treatment shifts the GSDM from a pro-tumoral role to an anti-tumour pyroptotic function (114).

In terms of the gasdermins, GSDMC is the least well understood in the context of bacterial pathogenesis. There are some data suggesting GSDMC may play a role in skin immunity. GSDMC has been shown to play a role in MMP-1 expression in skin keratinocytes in the context of UV light exposure (115). While MMP and ECM proteins can be exploited by pathogens to promote invasion and infection (as reviewed in (116)) it is not clear if pathogens can exploit GSDMC to modulate MMP-1 expression in the skin. There is also evidence that the intravenous delivery of an immunotherapy based on *Listeria monocytogenes* encapsulated in red blood cell membrane was able to drive GSDMC-mediated pyroptosis in the tumour, successfully dampening the suppressive environment of the tumour (117). Although further work needs to be done to understand the key drivers of these processes, this is an interesting concept of exploiting bacteria-gasdermin interactions in tumour therapy, as has been covered elsewhere (118).

Outside of bacteriology, there are studies concerning the role of GSDMC in response to helminth infection, which suggests a role for GSDMC in gut immunity. Here, IL-4 and IL-13 induced typical type 2 responses as expected, but also induced the upregulation of GSDMC in gut organoid models (119). Moving to an *in vivo Nippostrongylus* infection model, there is enhanced lytic cell death and evidence of a p30 band associated with activated GSDMC in enterocytes. In a similar vein, Zhao *et al.* show that downstream of helminth infection the release of IL-33, an important alarmin for inducing type 2 responses, was GSDMC dependent in gut epithelium, and was induced by the O-linked N-acetylglucosamine modification of STAT6 (120). Together this evidence puts GSDMC central to anti-helminth immune response and controlling Type 2 immune response. Furthering our understanding of GSDMC’s role in gut inflammation, both infection or autoimmunity driven may provide novel approaches to gut pathology and insight enabling strategies to target GSDMC in disease (119, 120).

## GSDME

DFNA5, or now more commonly known as GSDME, is another pore forming family member of the gasdermin family, but with marked divergence in expression patterns (121). Unlike its cousins, GSDME is expressed in the heart, brain, placenta, and kidney. Evolutionary speaking GSDME is well conserved and is more aligned to its non-pyroptotic partner PJVK and has homologs in coral and teleost species, where it has a clear anti-bactericidal activity (122–124). Before the emergence of the pyroptotic function of gasdermins, GSDME was linked to a hearing loss disorder associated with a mutation that results in a lack of the C-terminal domain (90, 122). There are also relationships between GSDME, p53 and cancer progression, as well as methylation of the GSDME gene that influences metastasis in breast cancer and p53 stabilisation in colorectal cancer (125–127). More recent evidence has also implicated GSDME in photoreceptor degeneration, IBD, RA and virus-induced pathology (128–131).

Activation of GSDME is mediated by caspase-3 during apoptosis, and in response to chemotherapeutic agents, virus or death receptor driven apoptotic signalling (132, 133). Intriguingly, the rheostat for cell committal to apoptosis or caspase-3 mediated-pyroptosis seems to be determined by the cellular expression of GSDME. Depending on the level of GSDME the cell will either commit to apoptosis driven by activation of caspases as per usual, or if given the opportunity with an abundance of substrate, caspase-3 will drive GSDME-mediated pyroptosis, or as traditionally known secondary necrosis. In GSDMD null cell systems, caspase-4/5 driven non-canonical inflammasome signals are completely abrogated; however, in canonical inflammasome signalling, GSDME can act as a back-up in the absence of GSDMD, largely through the recruitment of caspase-8 (and subsequent activation of caspase-3) by ASC, or activation of caspase-1 by caspase-3 (19). This balance does seem to rely somewhat on the expression level of GSDME, and likely other contributing factors such as caspases and NINJ1, as to whether there is a built-in redundancy to the system (130, 134, 135).

In the context of bacterial pathology, there are examples of GSDME influencing bacterial invasion and survival. As above, this depends on the expression profile of the infected cell, and it would be interesting to understand the balance of GSDMD versus GSDME expression patterns relative to likelihood of encountering a pathogen. Interestingly, there is some evidence that GSDME function is dependent on expression level that controls sublytic cytokine release versus full lytic death response (136). There is a dependence on the caspase-1/3/8 driven activation of GSDME in the context of NLRP3 activation or, in *Salmonella* infection, through NLRC4 (136). In another example, the Gram-negative bacterium *Brucella* presents lipoproteins that initiate unique cascades, of which only one terminated in GSDME activation (137). The two major lipoproteins, L16 and L19, both induce typical pro-inflammatory cytokine release but only L19 can induce IL-18 secretion. Upon further investigation L16 could only drive pro-IL-18 production, rendered so by an upregulation of caspase-3/8. Whilst L19 could cause phosphorylation of X-linked inhibitor of apoptosis (XIAP), thereby inhibiting caspase-3 function, L16 could not and instead activated caspase-3, GSDME formation and pyroptosis of the Thp1 cells used in this model (137).

In addition to immune cells, GSDME also plays a role in colonic epithelial cells in response to *Campylobacter jejuni*, the bacteria responsible for the majority of foodborne gastroenteritis globally (138). One of the key virulence factors involved in *C. jejuni* infection is cytolethal descending toxin (CDT), which is also present in several other bacteria strains including *E. coli* and *Helicobacter pylori* (138). CDT can drive a number of cell-specific responses including caspase-9/3 activation in colonic epithelial cells, which subsequently activates GSDME to drive cell death, with dependency on oxidative stress. This observation was independent to caspase-8 and caspase-1 and, given the role of caspase-9 and ROS as shown by addition of NAC in abrogating the effect, it would be interesting to understand the role of GSDME in release of cytochrome c from the mitochondria in this setting, to activate the apoptasome and caspase-9. GSDME is also involved in periodontitis in response to *Fusobacterium nucleatum*, a common oral bacterium (139). In this case, *F. nucleatum* was able to skew macrophages to a proinflammatory state, accompanied by elevated levels of ZBP1 and GSDME-mediated pyroptosis, which was reduced by shutting down ZBP1. Interestingly, understanding the key regulatory mechanisms of GSDME and associated cellular death is important in deciphering host-pathogen relationships in common inflammatory conditions (139) as well as in providing the relevant information to enable therapeutic targeting of GSDME in these conditions.

Albeit not bacteria, a recent study highlighting GSDME in combating *Candida* infection is worth noting for the involvement of a subset of Th17 cells (140). In this case, these Th17 cells can utilise NLRP3-caspase-8-caspase-3 to drive IL-1α production and secretion, which is dependent on GSDME. GSDME transcript is upregulated in response to Th17 stimuli (TGF-β and IL-1β) but not Th12 (Th1) or Th4 (Th2), and GSDME possesses binding sites for Th17-associated RORα and BATF, whereas GSDMD was not influenced by any T cell cytokines. Much like the above examples, Th17 cells do not progress to full pyroptosis and instead sit at a sublytic level driving GSDME-mediated release of IL-1α. Critically however, this function of Th17s is fundamental for their ability to protect against fungal pathogens, namely *Candida*, yet *Staphylococcus* nor polyclonal TCR activation drove GSDME-mediated IL-1α production. Whether this is an intentional restriction to this clone, or a caveat of the system used is unclear, but it will be interesting to see whether GSDME-mediated release of IL-1α and other cytokines in additional model systems plays an important physiological role *via* similar cell types. Th17 cells have a well appreciated pathogenic role in many autoimmune diseases so understanding this further will also be important for defining the role of GSDME in Th17-driven pathology.

## PJVK/GSDMF

PJVK is the only gasdermin family member lacking a N-terminal domain and so cannot induce N-terminal pyroptosis, at least in the systems tested (10). There are links again to hearing disorders in humans and equivalent phenotypes in *Gsdmf* ^-/-^ mice (141–143), but the relevance of PJVK to cell death and immunity is unclear. Saying this, there is considerable similarity between PJVK and GSDME, albeit without the pore forming ability. Despite the similarity, one major difference in PJVK compared to other gasdermins is the presence of a small C-germinal zinc-finger domain with no known role or function. Given the divergence of GSDMF from its relatives it will be interesting to see whether this gene retains any similar functions to other gasdermins away from pyroptosis, or instead has taken on a new function of its own.

## Pharmacological development of GSDM molecules

The development of tool molecules is required early in target validation to probe the function of a protein and provide insights into its mechanistic and phenotypic roles in biology and disease phenotypes. Tool molecules are generally potent, selective small molecules that demonstrate cellular target engagement and have a clearly defined mechanism of action (MoA) (144–146). To ensure all these criteria are met, tool compounds must be quality controlled and thoroughly profiled. If they are not, observed phenotypes cannot be confidently linked to the biological target, leading to spurious conclusions.

Our knowledge of the GSDM family of proteins and their roles in various pathological pathways, whilst expanding, is hampered by an absence of high-quality tools. Efforts into the discovery of tool compounds for the GSDM protein family have primarily focussed around pyroptotic executor protein GSDMD, due to the reasons explained above in assay development and clear cellular endpoints. Going forward, utilising the more recent observations in other gasdermin family members may provide new avenues of molecule discovery. The inhibition of GSDMD, alike the other members of the GSDM family, could occur *via* numerous mechanisms. However, it was Liu et al. that discovered the key role C191 plays in N-terminal (NT) GSDMD oligomerisation and subsequent ability to form pores in cell membranes (41). While investigating NT-oligomerisation, an immunoblot study was carried and NT-GSDMD was placed under reducing and non-reducing conditions. The monomeric and oligomeric forms were observed under non-reducing conditions and only the monomeric form under reducing conditions. This suggested that NT-oligomerisation involved disulphide cross-linking. Following these results, individual mutagenesis of all cysteine residues to alanine on murine GSDMD revealed that C39A and C192A impaired GSDMD oligomerisation. These residues correspond to human C38 and C191. This work uncovered a characteristic of GSDMD that could be leveraged to provide a viable strategy to target inhibition of GSDMD pore formation. Furthermore, amongst the GSDM family members this characteristic has only been reported for GSDMD, providing an opportunity to selectively engage one GSDM protein while sparing other family members. This is important in the context of findings from Huang et al. that implicate GSDMA in proper epidermal differentiation and cornification in skin barrier maintenance (147).

**Figure 2:**
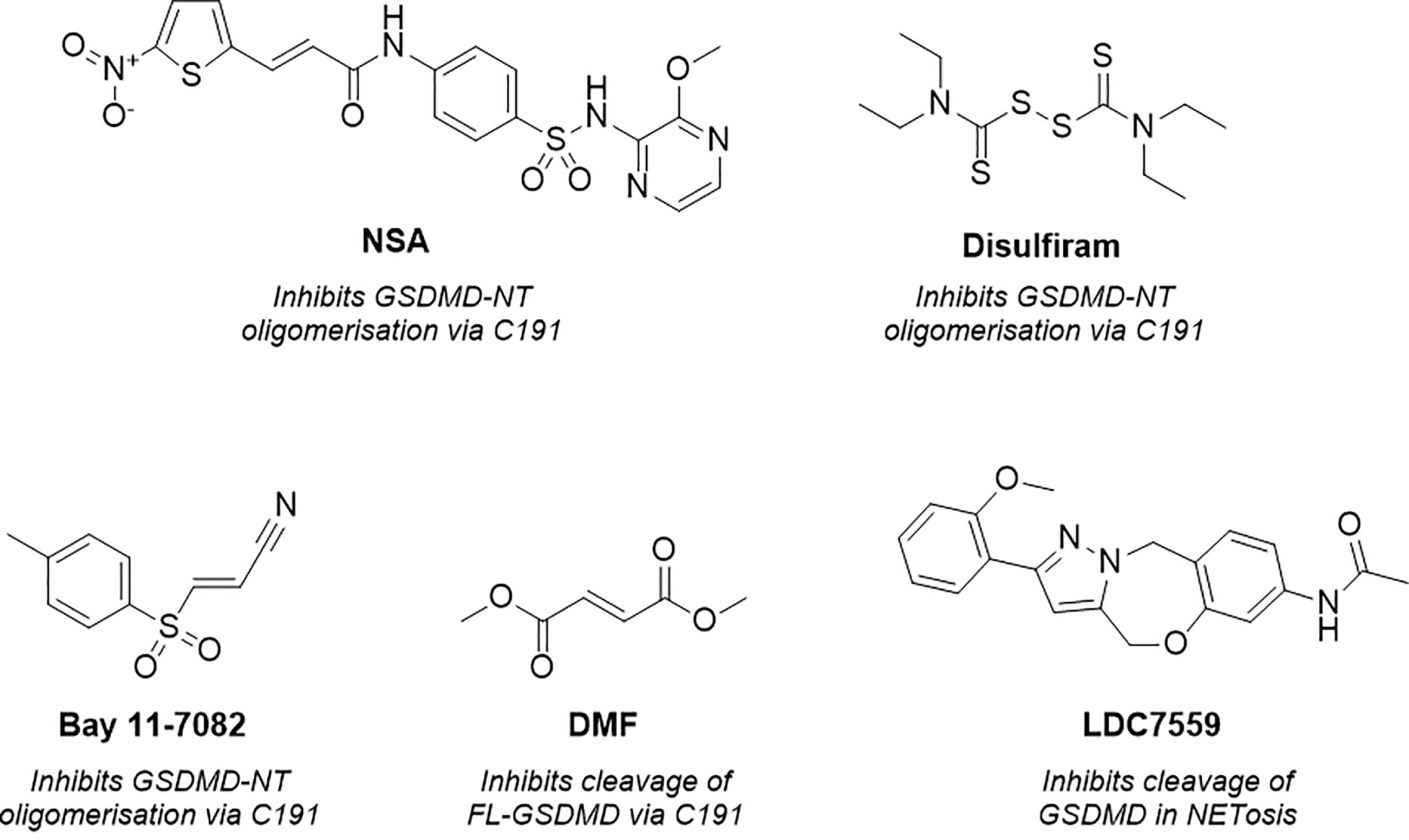
Structure of necrosulfonamide (NSA) (148), disulfiram, Bay 11-7082 (62, 152), dimethyl fumarate (DMF) (149) and LDC7559 (25), reported GSDMD inhibitors and their proposed mechanisms of action.

As a result of research by Liu et al. numerous tools were subsequently reported to prevent GSDMD pore formation *via* the covalent modification of C191 (62, 148, 149). Necrosulfonamide (NSA) (**Figure 2**) was the first of these (148). NSA had previously been identified as a MLKL inhibitor, a necroptosis mediator protein (150). The compound acted by modifying C86 of human MLKL, preventing MLKL polymer formation and subsequent necrotic cell death. Rathkey et al. hypothesised that NSA may act in a comparable manner with GSDMD (148). NSA was shown to prevent LPS-induced pyroptosis *in vitro*, and LPS-induced sepsis *in vivo*. NSA did not impair cleavage of the full-length (FL)-GSDMD to the NT, instead, a western blot study showed that NSA impaired NT-oligomerisation. GSDMD inhibition was believed to be *via* direct binding to C191, as reported through precipitation experiments between NSA and C191A-mutated GSDMD. While these experiments indicated that NSA prevented pyroptosis *via* covalent modification of GSDMD, useful mass-spectrometry (MS) based experiments would have installed further confidence and confirmed the proposed MoA. Furthermore, later studies revealed that NSA inhibited the activation of inflammasome NLRP3, caspase-1, and IL-1β, key components of the pyroptotic pathway (151).

Concurrently, Hu et al. identified GSDMD inhibitors disulfiram and Bay 11-7082 (**Figure 2**) in a small-molecule high-throughput screening (HTS) liposome leakage assay (62, 152). Disulfiram was the most potent compound identified and 22-fold more potent than Bay 11-7082 with an IC_50_ of 0.30 ± 0.01 µM in comparison to 6.88 ± 0.10 µM. NSA had also been screened in the liposome leakage assay and was the least potent of the three covalent binders with an IC_50_ of 9.28 µM ± 0.70 µM. To identify the site of covalent modification, disulfiram and Bay 11-7082 were incubated with FL-GSDMD. Following trypsin digest and MS analysis, C191 was identified as the major site of covalent modification. Both compounds were confirmed to inhibit pyroptosis in cellular studies where LPS-primed cells were treated with the compounds and cell death was observed by CyTox98 assay (62, 152). However, disulfiram and Bay-11-7082 have been shown to also inhibit inflammatory caspases through non-specific binding to their catalytic cysteine residues (62, 152). Furthermore, Bay 11-7082 is a widely reported NLRP3 inflammasome inhibitor (153).

More recently, while investigating the effect of Kreb’s cycle intermediates on pyroptosis, Humphries et al. reported a new covalent GSDMD inhibitor, dimethyl fumarate (DMF) (**Figure 2**) (149). LPS-induced pyroptosis was recovered upon treatment with DMF *in vitro*, this was also observed *in vivo* whereby treatment of DMF protected mice from LPS-induced shock. The small molecule, which succinates GSDMD cysteine residues, was shown to inhibit cleavage of FL-GSDMD, thereby preventing pore-formation, a notably different MoA to disulfiram and NSA. Incubation of DMF with purified FL-GSDMD, followed by trypsin digest, revealed that DMF covalently modified five cysteine residues, C56, C191, C268, C309, C467, where C191 was the major modified residue. It is worth noting that the covalent modification of C191 had not previously been linked to the inhibition of FL-GSDMD cleavage, solely the prevention of NT oligomerisation. It is therefore possible that the MoA observed is the product of covalent modification of one of the remaining cysteine residues, such as C268, a residue located in the flexible linker region of FL-GSDMD in proximity to the site of caspase cleavage. Nonetheless it would be interesting to investigate whether DMF also impaired the oligomerisation of NT-GSDMD, utilising the non-reducing western blot conditions described by Rathkey and Hu et al. for disulfiram and NSA (62, 148). It is also worth highlighting that the multiple covalent binding events observed is indicative of non-specific binding to GSDMD, with covalent modification being driven by intrinsic reactivity, and as such, promiscuity is to be expected. DMF was also included in an ABPP-proteomics experiment carried out by the Cravatt Lab (154). DMF did not significantly enrich any GSDMD peptides, demonstrating limited cellular engagement with GSDMD. The *in vitro* and *in vivo* profile described by Humphries et al. is therefore likely driven by interactions with several additional proteins in the pyroptotic cascade.

Finally, as observed with the aforementioned covalent tools, LDC7559 (**Figure 2**) was also initially reported to prevent NETosis by reversibly inhibiting GSDMD, as covered above (25). This was disproven in various cellular and recombinant protein assays by Amara et al., whereby LDC7759 was unable to recover LPS induced pyroptosis, in addition to failing to prevent GSDMD pore formation in a liposome leakage assay (62).

Numerous covalent and reversible tools for GSDMD have been reported in the literature. Whilst initial investigation into these tools appeared promising, characterisation was insufficient. Further studies reported a lack of selectivity to GSDMD, with numerous off-targets within the pyroptotic pathway described. Furthermore, the exact manner in which GSDMD is inhibited was poorly understood. As previously described, both factors are key for instilling confidence in a tool and any observed phenotypic changes. As such, higher quality tools for GSDMD investigation, and the wider GSDM family, are needed. The findings highlighted above noting C191 as a key residue in regulating GSDMD-mediated pyroptosis provide important groundwork in directing the discovery of more selective GSDMD tool inhibitors using reactive compound libraries.

Moving forward, compounds that are found to engage a GSDM protein must therefore be fully characterised to ensure the *in vivo* profile aligns with the *in vitro* and recombinant profile. Western-blot studies have proven useful to investigate potential MoA, with antibodies available for all GSDM proteins. The GSDMD liposomal leakage assay reported by Hu et al. should be transferable to the broader GSDM proteins and is another powerful tool to assess the efficacy and potency of GSDM inhibitors (62). Where covalent tools are investigated, mass-spectrometry workflows should be utilised to characterise covalent binding to the GSDM protein of interest, and the broader proteome using chemoproteomics in a cellular setting. It is worth noting that tool optimisation and discovery is limited by the absence of available crystallography for these pore-forming proteins. Indeed, due to the disordered linker region, entirely resolved crystal structures have thus far not been obtained. GSDMD C-terminal and a partial FL-GSDMD is currently accessible, however the linker region which contains the key C191 residue is absent. As such, the discovery and optimisation of tool compounds will need to be ligand-driven and through screening by HTS and fragment screening until further structures are defined. We await additional molecular and biophysical findings as discussed throughout this review that will allow novel more specific strategies to target the various GSDM in host defence and immune-mediated diseases.

## Summary

Since 2015 the gasdermin protein family have been front and centre for innate immunological research. As the field expanded beyond the discovery of GSDMD in pyroptosis, we have witnessed new influencers of GSDMD biology, alternative roles for gasdermins beyond that of pore formation, how GSDMA, GSDMB, GSDMC and GSDME are modulated and, most importantly, how gasdermins help protect us against pathogen invasion, and how pathogens have fought back *via* evolution. Furthering our insights into the pathogen-gasdermin relationship will be crucial to appreciating where gasdermin-based therapies could be efficacious. In the context of bacterial pathogens which evade detection by blocking inflammasomes, transient enhancement of specific protective GSDMD-mediated functions such as appropriate mitochondrial targeting may be beneficial. In contrast, immune-mediated diseases which are augmented by GSDMD activity such as sepsis and Familial Mediterranean Fever (FMF) may benefit from blockade of GSDMD-mediated pore formation. Interestingly, recent work from Jorch et al. demonstrated GSDMD-dependent secretion of alarmins driving autoinflammation in FMF which further highlights the need for GSDMD inhibitors (155).

As gasdermins gather more traction and undoubtedly become even more integral to several immunological processes, the need for pharmacological tools as well as inhibitors for clinical use will become critical. We look forward to developments in this area as researchers overcome hurdles associated with gasdermin biology and assess how effective modulating gasdermin biology is in multiple indications. While we have journeyed far in expanding our understanding and appreciation of GSDMs in host defence and disease, the endgame is not yet near.

## Author contributions

CG, MW-D, AB and LB collectively researched and wrote this article. All authors contributed to the article and approved the submitted version.
